# Biotransformation of acetophenone and its halogen derivatives by *Yarrowia lipolytica* strains

**DOI:** 10.1007/s13213-014-0955-3

**Published:** 2014-08-22

**Authors:** Tomasz Janeczko, Wojciech Bąkowski, Ewa Walczak, Małgorzata Robak, Jadwiga Dmochowska-Gładysz, Edyta Kostrzewa-Susłow

**Affiliations:** 1 Department of Chemistry, Wrocław University of Environmental and Life Sciences, Norwida 25, 50-375 Wrocław, Poland; 2 Department of Biotechnology and Food Microbiology, Wroclaw University of Environmental and Life Sciences, Norwida 25, 50-375 Wrocław, Poland; 3 Department of Medicine, The Witelon University of Applied Sciences, Sejmowa 5A, 59-220 Legnica, Poland; 4 Department of Cosmetology, Wrocław College of Physiotherapy, Kościuszki 4, 50-038 Wrocław, Poland

**Keywords:** *Yarrowia lipolytica*, *SUC2*, *URA3*, Enantiospecific reduction, Halogen derivatives of acetophenone

## Abstract

The ability of 16 strains of *Yarrowia lipolytica* to biotransform acetophenone and its derivatives has been studied. Thirteen of these strains were derived from a wild-type strain *Y. lipolytica* A-101; six had the invertase gene (*SUC2*) from *Saccharomyces cerevisiae* integrated into their genome, as well as the damaged or undamaged gene encoding orotidine-5′-phosphate decarboxylase (*URA3*), three had integrated the damaged *URA3* gene into their genome and three were UV acetate-negative mutants, not able to growth on acetate as the sole carbon source. The other tested strains included two wild strains, A-101 and PMR-1, and an adenine auxotroph ATCC 32-338A. All strains were capable of reducing acetophenone to the *R*-alcohol in high enantiomeric excess (80–89 %). In all of the cultures tested, reversibility of the reduction was observed, which led to an increase in the enantiomeric excess. nantioselective reduction of the acetophenone halogen derivatives revealed that the nature and location of the halogen atom had a significant influence on the enantioselectivity of the reduction. In the culture of ATCC 32-338A, after a 3-day biotransformation of 2,4′-dibromoacetophenone the enantiopure *R*-alcohol was obtained at a rate of 100 % of substrate conversion. In conclusion, using these invertase-containing strains or uracyl auxotrophs provided no additional benefit in terms of biotransformation capacity over the parental strain.

## Introduction

*Yarrowia lipolytica* is a dimorphic yeast that forms cream-colored, wrinkled, rough or smooth surfaced colonies (Domínguez et al. 2000; Perez-Campo and Domínguez 2001; Ding et al. 2004). Strains of this species have been isolated from margarine, cereal plants, high-protein meat products, vegetables, frozen food, wines, tar and petroleum-contaminated soil (Wojtatowicz et al. 1993; Alkasrawi et al. 1999; Deak et al. 2000; Thevenieau et al. 2009; Robak et al. 2011). This yeast can grow on many nutrients, including glucose, fructose, organic acids, alcohols, acetate or hydrophobic substances, such as fatty acids and alkanes (Wojtatowicz et al. 1991; Ravindra 2000), but growth is thiamine-dependent. It has outstanding physiological and biochemical properties, mainly due to the ability to use unconventional carbon sources and the secretion of extracellular hydrolases. *Y. lipolytica* is capable of growth on waste substrates, such as hydrolyzed molasses, spent sulfite liquor and raw glycerol, and therefore can qualify as a producer of cheap single cell biomass (SCB). This biomass is a valuable source of vitamins, exogenous amino acids and unsaturated fatty acids for animal feed (fodder yeasts) (Juszczyk et al. 2005). Moreover, in terms of human health, yeast proteins contain more of the exogenous amino acids (lysine, leucine, methionine) than algal and plant proteins (Ravindra 2000). Because of its proteolytic and lipolytic activities, this yeast is used in cheese production, particularly in the ripening process (Wojtatowicz et al. 2001; Lanciotti et al. 2005; Czajgucka et al. 2006).

*Yarrowia lipolytica* is capable of synthesizing lactones through the transformation of fatty acids (Pagot et al. 1998). One of the products is gamma-decalactone of peach aroma, produced from castor oil (Wojtatowicz et al. 1991; Fantin et al. 2001). Other valuable products are organic acids, such as citric, isocitric, oxoglutaric and pyruvic acids (Morgunov et al. 2004, 2013; Finogenova et al. 2005; Thevenieau et al. 2009) and erythritol (Tomaszewska et al. 2014; Mirończuk et al. 2014). Therefore, it is a species of great biotechnological importance in biosynthesis, biodegradation and biotransformation processes (Bankar et al. 2009; Coelho et al. 2010; Robak et al. 2011; Żogała et al. 2012; Rywinska et al. 2013).

Nowadays *Y. lipolytica* is categorized as a hemiascomycetous yeast, however phylogenetically it is distantly related to *Saccharomyces cerevisiae* (Sherman et al. 2004; Richard et al. 2005; Acker et al. 2008; Mekour et al. 2010; Naumova et al. 2010). The genome of *Y. lipolyica* was sequenced 10 years ago (Casaregola et al. 2000; Sherman et al. 2004) and unusual characteristics were found. The most important of these are its size (20.5 Mbp, twice the size of the *S. cerevisiae* genome), considerably high contents of G+C (49 %), the presence of 1,119 introns, chromosomal features of atypical structure (centromers and replication origins), a large number of tRNA genes (510), dispersed 5S rRNA genes (109 copies) and a novel organization of RNA genes (Acker et al. 2008; Mekour et al. 2010). The focus of much current research is the the study of new aspects of gene expression regulation (Neuveglise et al. 2005; Mekour et al. 2010; Blazek et al. 2011), which has resulted in the description of new phylogenetically related species (Knutsen et al. 2007; Michely et al. 2013).

*Yarrowia lipolytica* metabolizes only a few monosaccharides, namely, glucose, fructose, mannose and ribose. It cannot utilize polysaccharides and disaccharides, such as sucrose or lactose (Madzak et al. 2004; Morgunov et al. 2004; Rywinska et al. 2013). Since the 1980s there have been a persistent effort to effect the genetic improvement and development of *Y. lipolytica* strains, with a focus the efficient production of heterologous proteins or organic acids (Gasmi et al. 2001, 2011; Nicaud 2012).

One relatively easy approach to select potential new producers can be selection based on the ability to metabolize a non-typical (for the species under study) source of carbon, i.e. sucrose. Sucrose undergoes hydrolysis to glucose and fructose in the reaction catalyzed by an invertase, the enzyme encoded by the *SUC*2 gene in *S. cerevisiae.* Strains of *Y. lipolytica* containing invertase (Suc^+^) have been obtained by Nicaud et al. (1989), Förster et al. (2007) and Walczak and Robak (2009). Due to their capacity to grow on sucrose, recombinant strains of *Y. lipolytica* can utilize sucrose-containing substrates, such as non-hydrolyzed molasses and table sugar (Nicaud et al. 1989; Li et al. 2012). *Y. lipolytica* transformants with the Suc^+^ phenotype differ by various metabolic properties from the parental strain (Lazar et al. 2011, 2013; Michely et al. 2013) and therefore can be regarded as having a novel biotransformation ability.

Growing interest in the synthesis of enantiomerically pure compounds has promoted a great development in biocatalysis. Biotransformation is a convenient method for preparing chiral compounds. Chiral alcohols have been prepared through the reduction of aromatic ketones using yeast, fungi, bacteria, plants and isolated enzymes as biocatalysts. Enantiopure alcohols are useful chiral building blocks in the asymmetric synthesis of bioactive compounds of complex structures (Linder et al. 1989; Yadav et al. 2001; White et al. 2006; Janeczko et al. 2013). For this reason, studies on effective ways of obtaining these compounds on a preparative scale have been undertaken. The asymmetric reduction of ketones with the help of biocatalysts is a method that fulfills green chemistry requirements (Nakamura et al. 2003). In recent years several reports on the employment of isolated dehydrogenases for enantiospecific reduction have appeared (Zhu et al. 2009; Hoff and Sundby 2013; Xu et al. 2013; Rodríguez et al. 2014). The alcohol dehydrogenase from the hyperthermophilic archaeon *Pyrococcus furiosus* (PFADH) effectively catalyzes the reduction of various substituted α-chloroacetophenones to form the (*R*)-enantiomers of the corresponding chlorohydrins with an excellent enantiomeric purity (Zhu et al. 2009; ). However, the employment of intact microorganism cells of both growing and resting-state cultures eliminates problems with the regeneration of co-factors cooperating with enzymes. The associated low costs and simplicity of the microbial reduction of the carbonyl group make this experimental procedure an interesting alternative to methods which use synthetic catalysts (Coelho et al. 2010).

As substrates for the tests reported here we have chosen α-halogen derivatives of acetophenone because products of their reduction are used as chiral synthons in the pharmaceutical industry. The examples used here are the syntheses of (*R*)-(−)-formoterol, (*R*)-(−)-isoproterenol, (*R*)-(−)-salmeterol, (*R*)-(−)-denopamine, d-sotalol and (*R*)-(−)-clorprenalin (Corey and Helel 1998; Goswami et al. 2001), which are important beta2-adrenergic receptor agonist drugs.

The aim of this study was to test 16 strains of *Y. lipolytica* for their ability to transform acetophenone and its halogen derivatives. Nine strains are new, derived from a wild-type *Y. lipolytica* strain A-101, by genetic transformation (Walczak and Robak 2009). With the help of gene engineering tool,s some of transformants were obtained by homologous integration into the genome of the SUC2 gene (clones Suc^+^ura^−^) and others by non-homologous gene integration (clones Suc^+^Ura^+^ and suc^−^ura^−^). One of the clones obtained had two copies of the invertase gene (Lazar et al. 2011). All of these strains could potentially manifest new biotransformation properties.

## Materials and methods

### Substrates and strains

The substrates used in this study are acetophenone (**1**), 2,4′-dichloroacetophenone (**3**), 2,2’,4′-trichloroacetophenone (**4**), 2-chloro-4′-fluoroacetophenone (**5**), 2-bromo-4′-chloroacetophenone (**6**) and 2,4′-dibromoacetophenone (**7**); all were purchased from Sigma-Aldrich (St. Louis, MO). All racemic alcohols (**2**, **8**–**12**) were prepared by reducing the corresponding ketone with sodium borohydride in methanol.

The strains of *Y. lipolytica* were obtained from the Department of Biotechnology and Food Microbiology, Wrocław University of Environmental and Life Sciences and included (1) A-101 (wild-type parental strain), (2) A-101-1.31 (acetate-negative UV mutant of A-101), (3) A-101-1.31-K1 (smooth segregant of acetate-negative UV mutant of A-101-1.31), (4) A-101-1.22 (acetate-negative UV mutant of A-101), (5) PMR-1 (isolated from soil contaminated by fuel) and (6) ATCC 32-338A (adenine auxotroph purchased from the American Type Culture Collection). Also tested were transformants of A-101: (7) A18 (Suc^+^ura^−^), (8) B54-6 (Suc^+^ura^−^), (9) B55-3 (Suc^+^ura^−^), (10) B14-6 (Suc^+^ura^−^), (11) B57-4 (Suc^+^ura^−^), (12) B1-1 (suc^−^ura^−^), (13) A50 (suc^−^ura^−^), (14) B9-2 (suc^−^ura^−^), (15) Klon1 (Suc^+^Ura^+^) and (16) B56-5 (Suc^+^Ura^+^).

Strains numbers 7–16 underwent genetic transformation by a yeast cassette carrying the *SUC2* invertase gene from *S. cerevisaie*, flanked by two ura3 sequence fragments, homologous to the native *URA3* gene, encoding orotidine-5′-phosphate decarboxylase from *Y. lipolytica*. Using the transformation procedure described by Walczak and Robak (2009), we obtained three groups of recombinants. The first group (#8–11) consisted of four strains recombined at the desired site, characterized by the Suc^+^ura^−^ phenotype. The second group (#12–14) contained suc^−^ura^−^ strains, in which the cassette was not fully exchanged and, therefore, these strains did not gain the ability to grow on sucrose and become uracil auxotrophs. The third group (#15, 16) were strains in which the DNA hybridization took place at a location other than the target location, giving the strains the Suc^+^Ura^+^ phenotype. *Y. lipolytica* transformant B56-5 has two invertase encoding genes (Lazar et al. 2011).

### Culture media

The YPG growth medium was prepared with yeast extract (10 g/l), peptone (20 g/l) and glucose (20 g/l) and was sterilized in an autoclave at 121 °C for 20 min. Thiamine (0.3 mg/l) and uracil (20 mg/l) were added after sterilization.

The minimal growth medium MMT containing sucrose was prepared by mixing the following ingredients in 100 ml of water: (NH_4_)H_2_PO_4_ (5 g/l), KH_2_PO_4_ (2.5 g/l), MgSO_4_ ⋅ 7H_2_O (1 g/l) and 1 ml/l of mineral salts [Ca(NO_3_)_2_  ⋅ 4H_2_O (2 g) , Na_2_MoO_4_ (20 mg), FeCl_3_  ⋅ 6H_2_O (200 mg), CuSO_4_  ⋅ 5H_2_O (10 mg), H_3_BO_3_ (50 mg), CoCl_2_ (10 mg), MnSO_4_  ⋅ 4H_2_O (40 mg), KI (10 mg), ZnSO_4_  ⋅ 7H_2_O (40 mg). After sterilization in an autoclave, sucrose (10 g/l), thiamine (0.3 mg/l) and uracil (20 mg/l) were added.

### Growth conditions

For the analytical tests the biological material stored on agar slants was transferred into 250ml Erlenmeyer flasks containing 50 ml of YPG or MMT medium with the appropriate additives. Then flasks with the cultures were then placed on a shaker (150 rpm) for 24 h at 25 °C.

For the preparative-scale biotransformations, yeasts cultures were cultivated in 2,000-ml Erlenmeyer flasks containing 500 ml of YPG medium with appropriate additives on a shaker (150 rpm) for 24 h at 25 °C.

### Screening procedure

For the analytical tests the cultures were centrifuged under aseptic conditions (21,460* g* for 10 min at 25 °C), and the biomass [number of cells ≈ 5 ×10^8^ (cell/ml)] was transferred into 250-ml Erlenmeyer flasks containing 50 ml of previously sterilized phosphate buffer (1/15 M, pH 6.47). After transferring the biomass to the buffer, 10 mg of a substrate dissolved in 1 ml of acetone was added. After 1, 3, 6 or 9 days of incubation at room temperature and continuous shaking (150 rpm at 25 °C), 10-ml samples of the transformation mixture were removed. All procedure were carried out under aseptic conditions in microbiological safety cabinets (TopSafe 1.5, BIOAIR) or laminar flow clean benches (BIO 48, FASTER). The samples were extracted with 10 ml of CHCl_3_, dried over MgSO_4_, concentrated in vacuo and analyzed by GC. All the experiments were repeated three times.

### Preparative biotransformation

For the preparative-scale biotransformation the substrates (200 mg of each of compounds dissolved in 2 ml of acetone) were added to the cultures cultivated in 2,000-ml flasks (each containing 500 ml of the culture). The biotransformation mixtures were incubated under the same conditions (150 rpm, 25 °C) for 48 h. The mixtures were then extracted three times with 300 ml of CHCl_3_, dried over MgSO_4_ and concentrated in vacuo. Transformation products were separated by column chromatography using silica gel (Kieselgel 60, 230–400 mesh; Merck & Co., Whitehouse Station, NJ) and a hexane/acetone mixture (3:1, v/v) as the eluent.

### Analytical methods

The course of both the biotransformation and oxidation processes were followed using thin layer chromatography (TLC), and the composition of each product mixture was determined by capillary gas chromatography (CGC). Analytical TLC was carried out on silica gel G (Merck) with different developing systems. Compounds were detected by spraying the plates with 1 % Ce(SO_4_)_2_ and 2 % H_3_[P(Mo_3_O_10_)_4_] in 10 % H_2_SO_4_. GC analysis was performed using a Hewlett-Packard 5890A (Series II; Hewlett-Packard Co., Palo Alto, CA) GC instrument fitted with a flame ionization detector (FID). A chiral capillary column [Chrompack WCOT Fused Silica (CP Chirasil-DEX CB)], 25 m × 0.25 mm × 0.25 μm, (Varian Inc., Lake Forest, CA, ) was used to determine the composition of the unreacted substrates and product mixtures. Nuclear magnetic resonance (NMR) spectra were recorded with a DRX 500-MHz Bruker spectrometer (Bruker Corp., Billerica, MA) and measured in CDCl_3_. Optical rotations were measured with an Autopol IV automatic polarimeter (Rudolph Research Analytical, Hackettstown, NJ).

The GC conditions were an injector temperature of 200 °C and a detector temperature of 250 °C. The temperature programs for enantiomeric resolution of particular compounds are as follows:

- 1-phenylethan-1-ol (**2**): 90 °C/1 min, 3 °C min^−1^ to 105 °C, 30 °C min^−1^ to 200 °C/5 min, *R*_t_ (*R*) 4.97 min, *R*_t_ (S) 5.42 min.
- 2,4′-dichloro-1-phenylethan-1-ol (**8**): 142 °C/1 min, 2 °C min^−1^ to 165 °C, 30 °C min^−1^ to 200 °C/5 min, *R*_t_ (*S*) 10.05 min, *R*_t_ (*R*) 10.69 min.
- 2,2′,4′-trichloro-1-phenylethan-1-ol (**9**): 165 °C/1 min, 2 °C min^−1^ to 178 °C, 20 °C min^−1^ to 200 °C/5 min, *R*_t_ (*R*) 6.84 min, *R*_t_ (*S*) 7.42 min.
- 2-chloro-4′-fluoro-1-phenylethan-1-ol (**10**): 140 °C/1 min, 2 °C min^−1^ to 150 °C, 20 °C min^−1^ to 200 °C/5 min, *R*_t_ (*R*) 4.24 min, *R*_t_ (*S*) 4.64 min.
- 2-bromo-4′-chloro-1-phenylethan-1-ol (**11**): 160 °C/1 min, 2 °C min^−1^ to 178 °C, 20 °C min^−1^ to 200 °C/5 min, *R*_t_ (*R*) 9.49 min, *R*_t_ (*S*) 9.93 min.
- 2,4′-dibromo-1-phenylethan-1-ol (**12**): 150 °C/1 min, 2 °C min^−1^ to 183 °C, 20 °C min^−1^ to 200 °C/5 min, *R*_t_ (*R*) 14.20 min, *R*_t_ (*S*) 14.73 min.

### Identification of products

The obtained products proved to be secondary alcohols. The locations and orientations of the newly formed hydroxyl groups were determined on the basis of chemical shifts and shapes of the CHOH signals in the ^1^H NMR spectra:

- (*R*)-1-phenylethan-1-ol (**2**) [*α*]_*D*_^20^ = +38.6° (*c* = 3.0 CHCl_3_) (79 % ee); {Omori et al. (2007) [*α*]_*D*_^20^ = +51°, 99 % enantiomeric excess (ee)}; ^1^H NMR (CDCl_3_) δ 1.47 (d, 3H, *J* = 6.5 Hz, C*H*_3_), 2.79 (s, 1H, O*H*), 4.79 (q, 1H, *J* = 6.5 Hz, C*H*–OH), and 7.14-7.21 (m, 5H, *H*–Ar).
- (*S*)-2,4’-dichloro-1-phenylethan-1-ol (**8**) [*α*]_*D*_^20^ = +27.2° (*c* = 3.45 CHCl_3_) (86 % ee); {Wei et al. (1998), [*α*]_*D*_^20^ = +44.2°, 96.6 % ee}; ^1^H NMR (CDCl_3_) δ 2.67 (s, 1H, −O*H*), 3.60 (dd, 1H, *J* = 11.3, 8.6 Hz, one of C*H*_2_), 3.71 (dd, 1H, *J =* 11.3, 3.5 Hz, one of C*H*_2_), 4.88 (dd, 1H, *J* = 8.6, 3.4 Hz, C*H*–OH), and 7.26-7.39 (m, 4H, *H*–Ar).
- (*R*)-2,2’,4’-trichloro-1-phenylethan-1-ol (**9**) [*α*]_*D*_^20^ = −26.1° (*c* = 1.0 CHCl_3_) (68 % ee); (Huang and Ying (2007), [*α*]_*D*_^20^ = −59.1°, 90-92 % ee}; ^1^H NMR (CDCl_3_) δ 2.82 (s, 1H, O*H*), 3.52 (dd, 1H, *J* = 11.3, 8.5 Hz, one of C*H*_2_), 3.87 (dd, 1H, *J =* 11.3, 2.8 Hz, one of C*H*_2_), 5.26 (dd, 1H, *J* = 8.5, 2.8 C*H*–OH), 7.31 (dd, 1H, *J* = 8.3, 2.2 Hz, *H*5’–Ar), 7.38 (d, H1, *J* = 2.2 Hz, *H*6’–Ar), and 7.58 (d, 1H, *J* = 8.3 Hz, *H*3’–Ar).
- (*S*)-2-chloro-4’-fluoro-1-phenylethan-1-ol (**10**) [*α*]_*D*_^20^ = +34.7° (*c* = 4.75 CHCl_3_) (80 % ee); {Nieduzak and Margolin (1991), [*α*]_*D*_^20^ = +51°, 97 % ee}; ^1^H NMR (CDCl_3_) δ 2.73 (s, 1H, O*H*), 3.61 (dd, 1H, *J* = 11.3, 8.7 Hz, one of C*H*_2_), 3.71 (dd, 1H, *J* = 11.3, 3.5 Hz, one of C*H*_2_), 4.88 (dd, 1H, *J* = 8.7, 3.5 Hz, C*H*–OH), 7.04-7.09 (m, 2H, *H*3’,5’–Ar), and 7.32-7.38 (m, 2H, *H*2’, 6’–Ar).
- (*S*)-2-bromo-4’-chloro-1-phenylethan-1-ol (**11**) [*α*]_*D*_^20^ = +14.3° (*c* = 1.2 CHCl_3_) (42 % ee); {Basavaiah et al. (2004), [*α*]_*D*_^20^ = +39.0°, 89 % ee}; ^1^H NMR (CDCl_3_) δ 2.69 (s, 1H, O*H*), 3.50 (dd, 1H, *J* = 10.5, 8.8 Hz, one of C*H*_2_), 3.61 (dd, 1H, *J* = 10.5, 3.4 Hz, one of C*H*_2_), 4.91 (dd, 1H, *J* = 8.8, 3.4 Hz, C*H*–OH), and 7.20-7.28 (m, 4H, Hz, *H*-Ar).
- (*S*)-2,4’-dibromo-1-phenylethan-1-ol (**12**) [*α*]_*D*_^20^ = −36.1° (*c* = 1.8 CHCl_3_) (100 % ee); {Basavaiah et al. 2004), [*α*]_*D*_^20^ = +33.8°, 96 % ee}; ^1^H NMR (CDCl_3_) δ 2.75 (s, 1H, O*H*), 3.49 (dd, 1H, *J* = 10.5, 8.7 Hz, one of C*H*_2_), 3.60 (dd, 1H, *J* = 10.5, 3.4 Hz, one of C*H*_2_), 4.88 (dd, 1H, *J* = 8.7, 3.4 Hz C*H*–OH), 7.24-7.28 (m, 2H, *H*3’,5’–Ar), and 7.47-7.54 (m, 2H, *H*2’,6’–Ar).

## Results and discussion

The aim of this research was to check the strains of *Y. lipolytica* with a known phenotype and genotype with respect to their ability to biotransform the prochiral mixed aliphatic–aromatic ketone acetophenone. The reduction ability of these strains was also evaluated. Because of the reversibility of the reduction observed in the latter experiment, biotransformations were also performed in a racemic mixture of 1-phenylethan-1-ol. Additionally, we studied enantioselective reduction of halogen derivatives of acetophenone in cultures of selected strains.

In order to check the influence of *SUC2* gene expression on the biotransformation process, experiments with biomass grown on MMT medium containing sucrose as the sole carbon source were also carried out.

### Enantioselective reduction of acetophenone (**1**) in cultures of selected strains

**Fig. 1:**
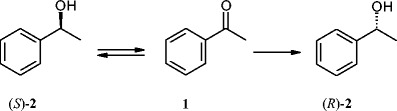
Reduction of acetophenone (**1**) to (*R*)-1-phenylethan-1-ol [(*R*)-***2***] and to (*S*)-1-phenylethan-1-ol [(*S*)-***2***)] by *Yarrowia lipolytica* strains

**Fig. 2:**
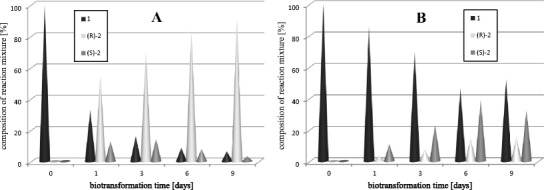
Time-course of biotransformation of acetophenone (**1**) by *Y. lipolytica* B55-3 (**a**) and *Y. lipolytica* ATCC 32-338A (**b**)

The ability of the parental strain *Y. lipolytica* A-101 and its derivatives (A18, B54-6, B55-4, B57-4, A50, B9-2, B56-5, B14-6, Klon1) to reduce acetophenone (**1**) was assessed. The results showed that genetic modifications only slightly influenced the yield of the reduction and the enantiomeric excess of the obtained alcohol (**2**) (Table 1). An efficient reduction of the substrate took place in all of the cultures, leading to the anti-Prelog product (*R*)-1-phenylethan-1-ol (**2**) with high enantiomeric excess. Due to the reversibility of the acetophenone reduction reaction in the cultures of the selected strains (Fig. 1), the highest enantiomeric excess (ee = 80–90 %) for (*R*)-alcohol (**2**) was achieved as many as 9 days after the biotransformation (Table 1). After 1 day of substrate incubation, the *R*-alcohol was obtained with 50–60 % of enantiomeric excess (data not shown). Our observations revealed that the longer the reaction time, the higher the predominance of this enantiomer, possibly explained by the oxidation of *S*-alcohol (**2**) back to the ketone (Figs. 1, 2). A similar tendency was observed for the *Y. lipolytica* strain ATCC 32-338A, where a gradual drop in *S*-alcohol content in relation to the *R*-alcohol (**2**) was noted. However, unlike the other tested strains, ATCC 32-338A produced predominantly (*S*)-l-phenylethan-1-ol (**2**), which is in accordance with the Prelog’s rule (Fig. 2).

**Table 1:** Biotransformation of acetophenone (**1**) by selected *Yarrowia lipolytica* strains (after 9 days)

Strain	Conversion (%)^a^	ee of alcohol **2** (%)
Wild strains		
PMR-1	91 ± 2	89 (*R*)
A-101	93 ± 0	83 (*R*)
Mutants		
A-101-1.22	92 ± 2	80 (*R*)
A-101-1.31-K1	87 ± 3	80 (*R*)
A-101-1.31	85 ± 4	73 (*R*)
ATTC 32-338A	48 ± 1	36 (*S*)
Clones		
A18	89 ± 3	82 (*R*)
B54-6	88 ± 4	88 (*R*)
B55-3	94 ± 2	93 (*R*)
Klon1	92 ± 1	89 (*R*)
B1-1	92 ± 4	92 (*R*)
A50	87 ± 4	88 (*R*)
B56-5	94 ± 1	81 (*R*)
B9-2	94 ± 2	90 (*R*)
B14-6	92 ± 1	79 (*R*)
B57-4	93 ± 0	83 (*R*)

ee, Enantiomeric excess

^a^Values reported as the mean ± standard deviation (SD)

### Biotransformations of racemic 1-phenylethan-1-ol (**2**)

**Fig. 3:**
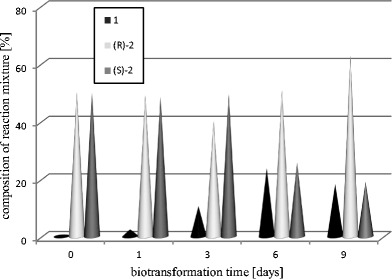
Time-course of biotransformation of 1-phenylethan-1-ol (**2**) by *Y. lipolytica* B9-2

The aim of the next experiment was to confirm that the reduction of acetophenone (**1**) is an equilibrium reaction. Therefore, a racemic mixture of 1-phenylethan-1-ol (**2**) was subjected to biotransformation. We observed a distinctive decrease in (*S*)-1-phenylethan-1-ol amount (**2**) and an increase in the *R*-enantiomer content of almost all tested strains after 6 (data not shown) and 9 days (Table 2) of the reaction. This result means that in the cultures of the tested strains the *S*-alcohol was oxidized to the ketone, and the formed acetophenone was reduced to *R*-alcohol **2**. The highest biotransformation yields were observed for *Y. lipolytica* strain A-101 with the suc^−^ura^−^ phenotype (A18 and B9-2). In the cultures of these strains *R*-alcohol **2** was obtained with the highest enantiomeric excess (Table 2; Fig. 3).

**Table 2:** Biotransformations of 1-phenylethan-1-ol (**2**) by selected *Y. lipolytica* strains (results after 9 days).

Strain	Percentage of alcohol determined by GC^a^	ee (*R*)^b^
A18	85 ± 2	49
B54-6	85 ± 3	26
B55-3	82 ± 0	51
klon-1	71 ± 5	3
B1-1	71 ± 3	25
A50	80 ± 1	52
B56-5	76 ± 5	15
B9-2	82 ± 1	54
B14-6	73 ± 1	36
B57-4	83 ± 2	36
A101	85 ± 4	19

GC, Gas chromatography

^a^Values reported as the mean ± SD

^b^Values reported as a percentage

On the basis of these results we selected the following strains for further experiments: parental strain *Y. lipolytica* A-101 and the strains B56-5 (Suc^+^Ura^+^), A18 (Suc^+^ura^−^) and A50 (suc^−^ura^−^).

### Biotransformation of acetophenone by *Y. lipolytica* A18 and B56-5 growing on sucrose

Due to the non-typical characteristic ability of *Y. lipolytica* to use sucrose as a carbon source, we cultivation all strains on a minimal growth medium MTM containing sucrose. The biotransformations were performed according to the procedure described in the section Screening procedure. The growth of the strain *Y. lipolytica* A18 was considerably inhibited (number of cells 3.2 × 10^7^ cells/ml) and, therefore, the results of the reduction were poorer (enantiomeric excess up to 71 %, but the conversion was only 23 % 9 days after the biotransformation). The strain *Y. lipolytica* B56-5 grew better and provided a much higher yield from the reduction (data not shown). The enantiomeric excess was comparable to the results obtained with yeast biomass growing on the rich culture media (YPG).

### Biotransformation of halogen derivatives of acetophenone (**1**) by the parental strain

**Fig. 4:**
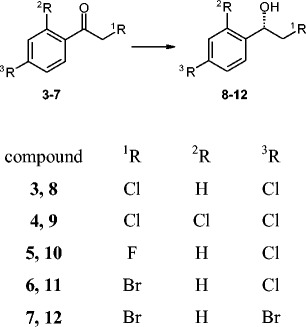
Halogen derivatives of acetophenone (**1**): 2,4′-dichloroacetophenone (***3***), 2,2′,4′-trichloroacetophenone (***4***), 2-chloro-4′-fluoroacetophenone (***5***), 2-bromo-4′-chloroacetophenone (***6***) and 2,4′-dibromoacetophenone (***7***). (***2***), (***8***–***12***) Racemic alcohols

We next checked the ability of the strain *Y. lipolytica* A-101 to reduce five halogen derivatives of acetophenone (**1**): 2,4′-dichloroacetophenone (**3**), 2,2′,4′-trichloroacetophenone (**4**), 2-chloro-4′-fluoroacetophenone (**5**), 2-bromo-4′-chloroacetophenone (**6**), and 2,4′-dibromoacetophenone (**7**) (Fig. 4).

**Fig. 5:**
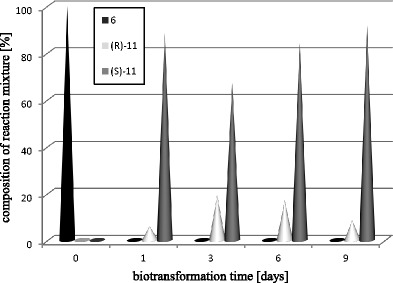
Time-course of biotransformation of 2-bromo-4′-chloroacetophenone (**6**) by *Y. lipolytica* A-101

It is worth noting that in the culture of *Y. lipolytica* A-101 the conversion was high (92–100 %) for all of the substrates used in this experiment. As with acetophenone (**1**), the majority of the substrateswere reduced to the anti-Prelog alcohol. Only for the substrate with the third chlorine atom at C-2′ (**4**) did we observe the opposite stereospecificity (Table 3). We also observed that the derivatives with bromine atom at C-2 (**6**, **7**) gave much lower enantiomeric excess (Fig. 4); for this group of substrates the reduction was reversible only for 2-bromo-4′-chloroacetophenone (**6**) (Fig. 5).

**Table 3:** Biotransformation of halogen derivatives of acetophenone (**1**) by selected strains of *Y. lipolytica*

Strain	Substrate^a^	Day	Conversion by GC (%)^b^	ee (%)	Configuration
B56-5	**3**	1	100	63	*S*
A18		1	99	68	*S*
ATCC 32-338A		1	20 ± 3	65	*R*
		9	27 ± 4	67	*R*
A50		1	58 ± 2	31	*S*
A-101		1	100	86	*S*
B56-5	**4**	1	89 ± 1	39	*R*
		3	100	42	*R*
A18		1	58 ± 2	31	*R*
		9	88 ± 1	47	*R*
ATCC 32-338A		1	24 ± 4	2	*S*
		3	62 ± 1	10	*S*
A50		1	57 ± 2	46	*R*
		9	100	48	*R*
A-101		1	100	69	*R*
B56-5	**5**	1	98 ± 1	68	*S*
A18		1	59 ± 3	54	*S*
		9	71 ± 3	53	*S*
ATCC 32-338A		1	11 ± 1	37	*R*
		9	12 ± 1	48	*R*
A50		1	39 ± 2	48	*S*
		9	55 ± 1	47	*S*
A-101		1	92 ± 1	80	*S*
B56-5	**6**	1	100	12	*S*
A18		1	100	2	*R*
ATCC 32-338A		1	100	74	*S*
A50		1	100	60	*R*
A-101		1	100	16	*S*
		9	100	55	*S*
B56-5	**7**	1	100	14	*R*
A18		1	100	9	*R*
ATCC 32-338A		1	64 ± 3	100	*R*
		3	100	100	*R*
A50		3	100	22	*R*
A-101		1	100	3	*S*

^a^(**3**), 2,4′-dichloroacetophenone; (**4**), 2,2′,4′-trichloroacetophenone; (**5**), 2-chloro-4′-fluoroacetophenone; (**6**), 2-bromo-4′-chloroacetophenone; (**7**), 2,4′-dibromoacetophenone

^b^Values reported as the mean ± SD

### Biotransformation of 2,4′-dichloroacetophenone (**3**) by selected strains derived from *Y. lipolytica* A-101

The tested strains of *Y. lipolytica* demonstrated different abilities to reduce 2,4′-dichloroacetophenone (**3**). *Y. lipolytica* B56-5 and A18, which possess the *SUC2* gene, showed properties similar to the parental strain and reduced the substrate with an almost 100 % yield and high (up to 70 % obtained after 3 days of transformation) enantiomeric excesses of the resulting *S*-alcohol (**8**) (Table 3). The lowest both reduction yield (60–80 %) and enantiomeric excess (up to 33 % 9 days after biotransformation) were observed for *Y. lipolytica* A-101 A50 (data not shown). In this case, the strain ATCC 32-338A also performed the reduction with the opposite enantiospecificity, producing a poor yied of *R*-alcohol (**8**) (not exceeding 30 %) (Table 3).

### Biotransformation of 2,2′,4′-trichloroacetophenone (**4**) by the selected strain of *Y. lipolytica*

The presence of the additional chlorine atom in the molecule of 2,2′,4′-trichloroacetophenone (**4**) explains why this compound is reduced to alcohol (**9**) with the opposite enantioselectivity, compared to the previous substrates. In this product, the *R*-enantiomer of alcohol (**9**) predominated. The results obtained are satisfactory; however, the conversion of the substrate is less efficient than that with the parental strain. For strain ATCC 32-338A, the conversion was much lower than that of the other tested strains, and the enantiomeric excess was also very low (the *S* enantiomer predominated) (Table 3).

### Biotransformation of 2-chloro-4′-fluoroacetophenone (**5**) by selected strains of* Y. lipolytica*

Similarly to the parental strain *Y. lipolytica* A-101, the derived strains demonstrated a high capacity for biotransforming 2-chloro-4′-fluoroacetophenone (**5**). The highest reduction yield, comparable to that of the parental strain, was observed for the clone with the Suc^+^Ura^+^ B56-5 phenotype. Strains A18 and A50 showed a lower substrate conversion (71 and 55 %, respectively). The parental strain and all clones gave predominantly the *S*-enantiomer of alcohol (**10**). The highest enantiomeric excess was noted for the parental strain (80 %) and clone B56-5 (68 %). A relatively lower enantiomeric excess was observed for strains A18 (54 %) and A50 (48 %). The *R*-enantiomer of alcohol (**10**) was obtained in the culture of ATCC 32-338A , but at a low substarte conversion (12 %) (Table 3).

### Biotransformation of 2-bromo-4′-chloroacetophenone (**6**) by selected strains of* Y. lipolytica*

The reversible reduction processes were observed (similarly to the parental strain) in the case of the biotransformation of 2-bromo-4′-chloroacetophenone (**6**) by the mutants of *Y. lipolytica* A-101. Due to changes in the composition of the reaction mixtures during incubation with the tested microorganisms, large differences among the results were noticed. The yields were high and the enantiomeric excesses were low. The parental strain produced *S*-alcohol (**11**) with an ee of 80 %, whereas strain A50, after a 1-day biotransformation, produced the *R*-enantiomer at an ee of 60 %. For the rest of the tested strains almost equimolar amounts of both enantiomers of (**11**) were obtained after 1 day of biotransformation. The most effective reduction of substrate (**6**) was achieved in the culture of strain ATCC 32-338A (Table 3).

### Biotransformtion of 2,4′-dibromoacetophenone (**7**) by selected strains of* Y. lipolytica*

Transformation of this substrate in the culture of strain ATCC 32-338A produced enantiopure *R*-alcohol (**12**) with a 100 % conversion rate (after 3 days of biotransformation). Among the strains tested, the highest enantiomeric excess (22 %) of the *R*-alcohol was observed in the culture of strain *Y. lipolytica* A50, after 3 days of biotransformation (Table 3). The parental strain and the other clones reduced the substrate with a high degree of conversion, but with low enantiomeic excess.

The asymmetric reduction of ketones is one of the most important reactions leading to chiral alcohols, which serve as substrates in the production of many drugs, vitamins and chemicals used in modern agro-technology (Goswami et al. 2001; Yadav et al. 2001; Huang and Ying 2007). The results of our study indicate that the applied genetic modifications did not have a strong influence on reactivity compared to the parental strain. However, our results are a good starting point for strain selection and for further optimization of biotransformation conditions, with the aim of increasing yields and obtaining optically pure products.

In order to check the ability of the tested strains to perform a reduction, a prochiral mixed aliphatic–aromatic ketone, i.e. acetophenone (**1**), was used as a model substrate. All of the mutants tested were capable of the reduction of acetophenone (**1**) to the *R*-alcohol with high enantiomeric excess. In all of the tested cultures reversibility of the reduction was observed, leading to an increase in enantiomeric excess. The reversibility of the reduction was studied by using racemic phenylethanol as a substrate for biotransformation by the selected strains. The result was a significant decrease in the amount of *S*-phenylethanol, leading to the *R*-alcohol with an ee of >50 %.

Our results indicate that neither targeted nor non-targeted genetic modifications have any strong impact on the yield and enantioselectivity of the enzymes responsible for the most common microbial transformations, such as the reduction and oxidation of xenobiotic substrates. Moreover, the clones of the Suc+ phenotype gained the ability to conduct biotransformations in growth media containing sucrose as a carbon source, which is not a typical characteristic of *Y. lipolytica* species.

Enantioselective reduction of acetophenone halogen derivatives was also performed. We observed that the nature and location of the halogen atom exerted a significant influence on the enantioselectivity of the reduction. A 3-day transformation of 2,4′-dibromoacetophenone (**7**) in the culture of ATCC 32-338A strain resulted in the production of the enantiopure *R*-alcohol (**12**) at a substrate conversion rate of 100 %. Such a good result with respect to the enantiomeric excess was unexpected because the other halogen derivatives were reduced by this strain much less effectively. This is a also notable result because this biotransformation gave no side products at all. In contrast, the production of additional side products have been observed in transformations of this substrate in cultures of other microorganisms (Rocha et al. 2010; Utsukihara et al. 2007).

Despite the fact that the strains of *Y. lipolytica* contain less selective alcohol dehydrogenases than some other biocatalysts catalyzing the enantioselective reduction of acetophenone and its halogen derivatives (Lin et al. 2009; Tokoshima et al. 2013), they do have a rare ability to reduce acetophenone to the anti-Prelog *R*-alcohol. However, the aim of our study, apart from presenting the capability of selected strains to perform the effective reduction, was to show differences in biocatalytic properties of strains within the same species. Moreover, we focused our attention to the reversibility of the reduction which takes place during biotransformation of the tested substrates in the cultures of *Yarrowia lipolytica*.
